# Construction of Zn^2+^ Chelated Dodecapeptide Assembled Hydrogel with Bio-Adhesive and Bone Regeneration Functions

**DOI:** 10.3390/gels12060511

**Published:** 2026-06-09

**Authors:** Jun Bai, Lenan Zhuang

**Affiliations:** Institute of Genetics and Reproduction, Department of Veterinary Medicine, College of Animal Sciences, Zhejiang University, Hangzhou 310058, China

**Keywords:** peptide assembled hydrogel, zinc chelating, bone regeneration

## Abstract

Hydrogels constructed from peptide components often rely on β-sheet architectures for their assembly, yet the process of developing such materials in aqueous environments presents notable hurdles in the context of biological systems. To address this, a novel functional dodecapeptide has been developed, capable of self-assembling into supra-molecular hydrogels via zinc chelation interactions. Morphological observations revealed a compact meshwork structure in the hydrogel formed with 9 mM Zn^2+^, differing from the relatively sparse or excessively tangled fiber architectures seen at other zinc concentrations. Alkaline phosphatase activity, an early marker of osteoblast differentiation, was notably enhanced when MC3T3-E1 cells were cultivated for 72 h in the hydrogel extract containing 300 μg/mL of the peptide, 9 μg/mL ZnCl_2_, and 18.93 μg/mL H_3_BO_3_. Furthermore, increased protein levels of p-p38/p38, p-ERK/ERK, and p-JNK1/2/3/JNK1/2/3 were observed in P-300-ZnB and P-300 B hydrogel-treated groups, suggesting an association with MAPK pathway activation. P-Zn-9 hydrogel also promoted MC3T3-E1 cell proliferation and demonstrated favorable biocompatibility in short-term in vitro and in vivo assays. Long-term toxicity and causal relationships via inhibitor studies remain to be investigated. These results offer a viable approach to endow zinc-chelating properties in the fabrication of assembled hydrogels, presenting an innovative and potential method for constructing injectable drug delivery systems and in situ bone repair through biomaterials in subsequent applications.

## 1. Introduction

Peptide-based supra-molecular hydrogels have garnered significant attention as a crucial class of biomaterials, finding applications in drug delivery systems, electronic device fabrication, biomedical fields, and bone tissue engineering [1,2]. Supra-molecular materials, which are constructed by molecular building blocks through non-covalent interactions like hydrogen bonds, electrostatic forces, and Van der Waals forces, can undergo colloidal transition or gelation from the liquid phase [3,4,5,6]. The capability of bone bio-adhesive materials to stimulate new bone growth and integrate with adjacent host tissues has garnered significant attention. In particular, nanostructured solvent-encapsulating systems based on metal ion-coordinated peptides can self-assemble into complex, higher-order supramolecular gels, offering a promising platform for bone repair applications [7,8]. Such systems, which frequently consist of solvents enclosed within nanostructures, can self-assemble into more complex and higher-order structural supra-molecular gels [7,9,10]. Metal ion-coordinated peptides, being a type of such system, typically allow for the design of organized structures with desirable properties induced by metal ions, thereby forming interconnected networks within a non-covalent system [11,12].

Bone tissue engineering represents an emerging discipline focused on developing bioactive constructs that can restore bone tissue functionality [13]. A primary objective in this domain is to replicate the body’s natural processes, encompassing self-repair mechanisms, structural adjustment, and the generation of new bone tissue [14,15]. Osteoblasts play a crucial role in the creation of new bone by synthesizing osteoid, a substance made up of collagen and various other proteins [13,14]. To optimize in situ bone formation, implant designs need to integrate bio-compatible and immunomodulatory properties, as these features can effectively support the regenerative process [15,16]. In conventional clinical applications, injectable bioactive glass materials have been widely employed for bone repair, demonstrating a relatively rapid ability to establish connections with surrounding connective tissues and form an amorphous calcium phosphate layer immediately after implantation [16]. However, recent advancements have shifted attention to injectable hydrogels, which are soft, biocompatible materials with high water content and adjustable mechanical properties. These three-dimensional networks, known for their ability to mimic the extracellular matrix, have gained widespread application in the domain of bioengineering, facilitating cell growth and bone repair processes. These three-dimensional networks, characterized by their high water content and adjustable mechanical properties, have garnered significant attention in biomedical research [2,17]. Hydrogels possess bio-compatibility, softness, and tunable architecture, allowing them to mimic the physical structure of the extracellular matrix to promote cell proliferation and bone regeneration [18,19]. Researchers have sought to create injectable hydrogels for tissue engineering, leveraging benefits like minimizing surgical procedures, conforming to irregularly shaped cavities, and establishing effective tissue adhesion [20]. 

Zinc ions play a crucial role in bone metabolism and regeneration. In particular, Zn^2+^-releasing hydrogel systems have been shown to exhibit antibacterial, immunomodulatory, and pro-osteogenic functions, highlighting their therapeutic potential for bone defect repair [21]. It is involved in more than 300 metalloenzymes and over 2500 transcription factors, highlighting its vital significance in biological systems [22]. However, some peptide-based hydrogels currently rely on organic solvents to form gels or toxic compounds for cross-linking, which significantly compromises their biocompatibility. Food-derived peptides, such as a dodecapeptide (IEELEEELEAER, P-2-CG) isolated from oysters and mussels, exhibit bioactive properties that facilitate bone regeneration [23,24]. Previous study systematically investigated the ability of various metal ions to induce peptide hydrogel formation. Compared with Ca^2+^, Mg^2+^, and Na^+^, Zn^2+^ was found to produce a peptide hydrogel with lower viscosity, suggesting a unique role for Zn^2+^ in modulating peptide assembly [25]. Building on this finding, the present work constructs a functional dodecapeptide (P-2-CG) capable of self-assembling into supramolecular hydrogels via zinc chelation. Distinct from previously reported zinc-chelated peptide systems that focused primarily on gelation behavior and structural characterization, our study further demonstrates, for the first time, the bio-adhesive properties of the Zn^2+^-induced hydrogel formed by the P-2-CG peptide on bone tissue and its capacity to promote bone regeneration in vivo. This advances the application of zinc-chelated peptide hydrogels from basic assembly studies toward potential biomedical uses in injectable bone repair. Osteoblasts, which are key cells in bone development, have the ability to release numerous osteogenic-active molecules, including growth factors, cytokines, and chemokines. These cells also play a role in regulating the maturation process of the extracellular matrix, ultimately contributing to the enhancement of bone formation. To assess the osteogenic effects of zinc-induced hydrogel formed by the P-2-CG peptide on osteoblasts, this research involved the culture of MC3T3-E1 cells to investigate their functional activities. To assess the osteogenic properties of zinc-loaded hydrogel formed by the P-2-CG peptide, the expression levels of osteoblast-related markers, such as alkaline phosphatase (ALP), the mRNA expression of runt-related transcription factor 2 (Runx-2), c-Jun N-terminal kinases 1/2/3 (JNK1/2/3), p38 mitogen-activated protein kinase, and extracellular signal-regulated kinase were measured in the hydrogel extracts. To meet the dual needs of injectable administration and bone repair, the supramolecular hydrogels developed in this work are believed to exert an influence on bio-adhesion, which arises not only from their ability to form a controllable 3D network but also from their capacity for zinc chelation assembly.

Unlike previous Zn^2+^-chelated peptide systems limited to gelation and structural characterization, this work first demonstrates the bio-adhesive property of a Zn^2+^-induced dodecapeptide hydrogel on bone tissue and its osteogenic capacity in vitro and in vivo. Building on prior comparative study of metal ions, this work advances the platform by integrating zinc chelation with injectable bio-adhesion and bone regeneration [25]. This conceptual shift from fundamental assembly to translational applications offers a new paradigm for food-derived peptide-based materials in bone tissue engineering.

## 2. Results and Discussion

### 2.1. Macroscopic Properties and Bio-Adhesive of Zinc Induced P-2-CG Hydrogel

**Figure 1:**
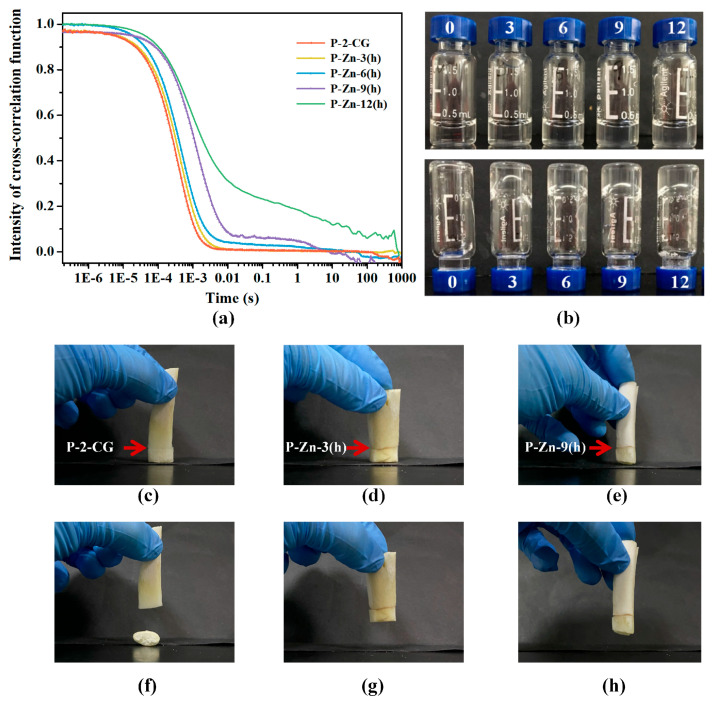
Macroscopic properties and bio-adhesive of P-Zn-9 hydrogel. (**a**) The intensity cross-correlation function detected by 3D DLS at indicated time scales of P-2-CG and different concentration of Zn^2+^ chelating peptides including P-Zn-3(h), P-Zn-6(h), P-Zn-9(h), and P-Zn-12(h). (**b**) Supra-molecular hydrogels formed by different concentrations of Zn^2+^ chelating peptides. Macroscopic properties of the supra-molecular structure transition from solution (upper image) to hydrogel (lower image) after 2 h incubated at 20 °C. Samples in each picture are P-Zn-0(h), P-Zn-3(h), P-Zn-6(h), P-Zn-9(h), and P-Zn-12(h) from left to right. (**c**–**e**)After P-Zn-0(h), P-Zn-3(h) and P-Zn-9(h) formed, an in vitro experiment was performed to verify the bio-adhesion of P-Zn-0(h) (**c**), P-Zn-3(h) (**d**), and P-Zn-9(h) (**e**) onto real bone tissue to simulate bone adhesion. (**f**–**h**) Vertical lifting test after 5 min of applied stress: (**f**) P-2-CG (control), (**g**) P-Zn-3(h), and (**h**) P-Zn-9(h).

Three-Dimensional Dynamic Light Scattering (3D DLS) studies were applied to investigate the assembly of peptide varied with different concentration of zinc. The cross-correlation functions of P-2-CG and zinc chelated hydrogels formed by P-2-CG including P-Zn-3(h), P-Zn-6(h), P-Zn-9(h), and P-Zn-12(h) were obtained from the intensities of the scattered light directly recorded (Figure 1a). Such a modulated 3D cross-correlation light scattering technique has high sensitivity, for an ideal DLS, the intensity of the auto-correlation function can be obtained with g^2^(t) of equal to 1, multiple scattering leads to a decrease in g^2^(t) due to the decreased correlation of light [26,27]. As indicated by He and Diener, the signal in the long relaxation time range corresponds to large aggregates, while the signal in the short relaxation time range corresponds to small aggregates [28,29]. Regarding the inapplicability of the Stokes-Einstein equation, the transformed results were not convincing for highly irregular particles, thus intensity of cross-correlation functions was used directly for these irregular particles. The curves of the cross-correlation function for P-Zn-6(h), P-Zn-3(h), and P-2-CG decreased sharply, while the curves for the peptides with higher zinc concentration samples including P-Zn-9(h) and P-Zn-12(h) decreased smoothly in Figure 1a. For samples with a high concentration of zinc, P-Zn-9(h) and P-Zn-12(h), the increased scattering indicated the formation of hydrogel. The transition from solution to hydrogel was presented in Figure 1b, as the system became jammed with the increase of zinc addition. A bone bio-adhesive material can be defined as a substance that, when applied between two bony surfaces, facilitates the bonding process. For example, protein and peptide components are desirable due to their inherent properties of biological recognition, favorable bio-degradability and bio-compatibility 5–9. After P-2-CG, P-Zn-3 and P-Zn-9 formed, a qualitative bone adhesion experiment was performed, two halves of rib bones were adhered using P-Zn-3 (Figure 1d,g) and P-Zn-9 (Figure 1e,h), as a control, bones coated with P-2-CG (Figure 1c,f) did not exhibit adhesive ability. These visual observations suggest that increasing the zinc concentration enables the formation of bio-adhesive hydrogels. However, quantitative measurements of adhesion strength are needed to further confirm this finding.

### 2.2. Micro-Structure of P-2-CG Hydrogel Induced by Zinc

**Figure 2:**
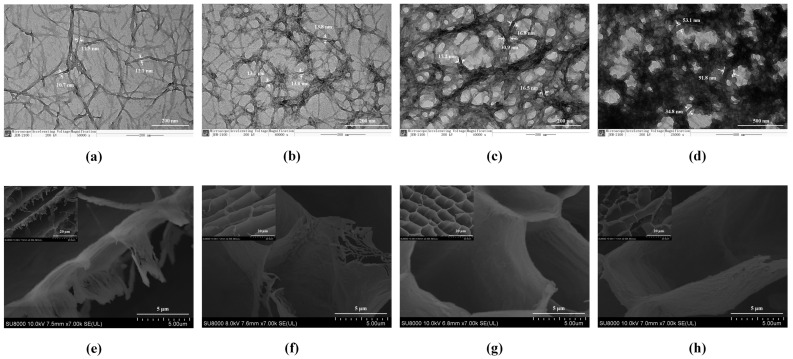
Morphology of assembled hydrogels. Cryo-TEM images of P-Zn-3(h) (**a**), P-Zn-6(h) (**b**), P-Zn-9(h) (**c**), and P-Zn-12(h) (**d**). Cryo-SEM images of P-Zn-3(h) (**e**), P-Zn-6(h) (**f**), P-Zn-9(h) (**g**), and P-Zn-12(h) (**h**) at low and high magnifications (2k× and 7k×).

The Cryo-TEM images revealed a fascinating transformation in the unordered arrangement of intertwined nano-fibrils, where these fibrillar structures tend to coil and wind around one another [30,31]. With the increasing concentration of zinc from 3 mM to 12 mM, clearly demonstrating the densification and thickening of the gel skeleton with increasing zinc concentration (Figure 2a–d). As shown in Figure 2a, P-Zn-3 fibrils with a screw pitch of 10.7–11.5 nm exhibited twists with a diameter approximately half to one-third of the pitch. More clusters of fibrils coiled together in P-Zn-9 (Figure 2c), formed a thicker skeleton, measyring about 16–17 nm and even 30.9 nm in screw pitch [31]. Skeletons are formed by clusters of fibrils, multi-stranded fibrils exhibit various structures at different length scales and cross sections, ultimately forming hydrogel networks [26,31]. Figure 2e–h show the Cryo-SEM images of P-Zn-3(h), P-Zn-6(h), P-Zn-9(h), and P-Zn-12(h) at low and high magnifications. The surface of the skeleton in P-Zn-9 was smoother and more flatter, it can be observed that the hole diameters between the cross-linked networks in P-Zn-9 were approximately 10 μm (Figure 2g). In comparison to other hydrogels including P-Zn-3(h), P-Zn-6(h), and P-Zn-12(h), which displayed either sparse or overly tangled fiber bundles, the distinct structural characteristics of P-Zn-9(h) were primarily characterized by its dense network architecture. The differences in size and network type suggest that the final hydrogel structure was influenced by the concentration of zinc. It facilitates the control of assembly and network formation, resulting in bio-adhesive properties for bone adhesion.

### 2.3. Molecular Structure Changes in P-2-CG Hydrogel Induced by Zinc

**Figure 3:**
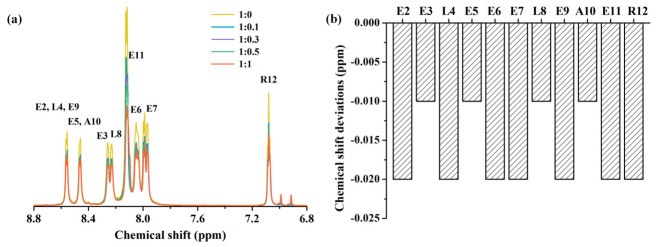
One dimensional ^1^H spectra and chemical shift deviations (CSD) plot of H values from P-Zn-0(h) to P-Zn-9(h). (**a**) One dimensional ^1^H spectra of 9 mM peptide P-2-CG as function of Zn^2+^ concentration from 1:0 to 1:1 (mol concentration of peptide/Zn^2+^). (**b**) CSD plot of H values from P-Zn-0(h) to P-Zn-9(h).

**Figure 4:**
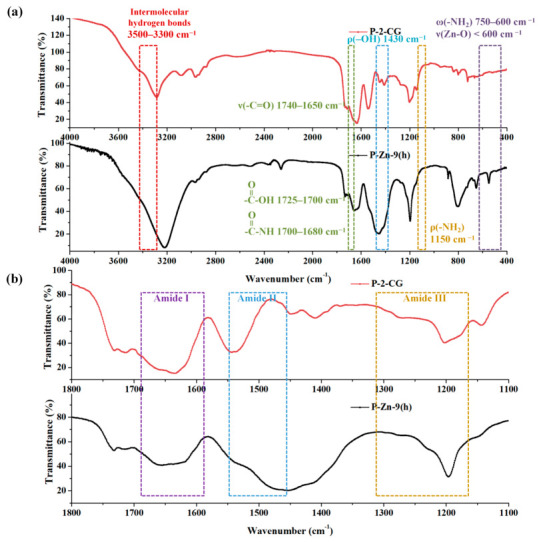
FT-IR spectrum of P-Zn-0(h) and P-Zn-9(h) from 4000 cm^−1^ to 400 cm^−1^ (**a**) and 1800 cm^−1^ to 1100 cm^−1^ (**b**).

**Figure 5:**
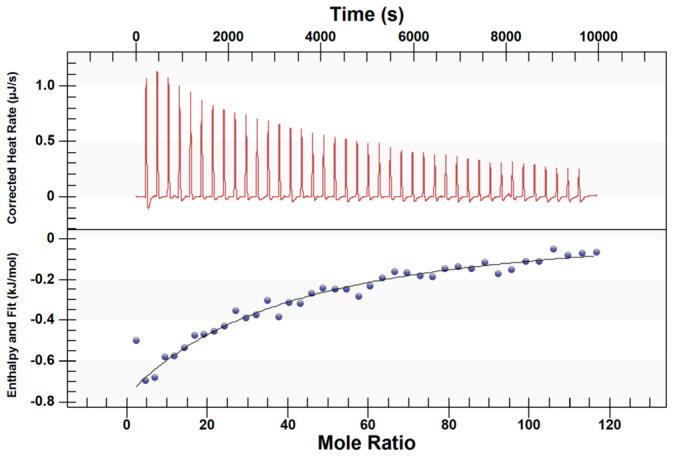
The calorimetric titration isotherms of the chelating interaction between Zn^2+^ and P-2-CG. The exothermic curve continuously decreased which indicated that 1.258 ± 0.208 mM Zn^2+^ bound to 1 mM P-2-CG. According to the fitting graph obtained by titration, the thermodynamic data of K_d_ (6.144 × 10^−3^ ± 1.502 × 10^−3^ M) showed that the peptide P-2-CG had a week affinity to Zn^2+^.

To monitor gel formation induced by zinc, one-dimensional ^1^H spectra of 9 mM P-2-CG were recorded as a function of zinc concentration ranging from 1:0 to 1:1 (molar concentration of peptide/zinc) over a 2 h period. The peak intensities of the amide protons arising from residual peptides in solution decreased with the increasing concentration of zinc (Figure 3), implying that more peptide molecules experienced structural transformation and participated in the hydrogel construction. To delve deeper into the reasons behind how zinc triggers structural transformation in the assembly, the shifts in absorption peaks observed via FTIR were analyzed, as these variations could indicate the interaction between zinc ions and potential binding sites on the amide/carbonyl groups present in P-2-CG [12]. The Amide A bands (3500–3200 cm^−1^) are important for assessing N-H stretching vibrations, which provided information on intermolecular hydrogen-bonding interactions (Figure 4). The Amide II band of P-2-CG shifted from 1453 cm^−1^ to 1538 cm^−1^ after Zn^2+^ chelation, indicating an alteration of the hydrogen-bonding environment around the amide groups. Similar shifts have been associated with changes in intermolecular hydrogen bonding involving the amide C=O and N–H groups, possibly coupled with contributions from side-chain hydroxyl groups [32]. Isothermal titration calorimetry (ITC), a common analytical technique, is frequently employed to investigate interactions occurring between various types of molecular systems, such as protein molecules, peptide chains, and other biological molecules, alongside small molecules like ligands, ions, and nucleic acids [33]. The exothermic curve continuously decreased, indicating that 1.258 ± 0.208 mM of zinc bound with 1 mM P-2-CG, which indicated the stoichiometry of zinc chelating to P-2-CGwas above 1 (Figure 5). According to the fitting graph, the thermodynamic data for Kd was 6.144 × 10^−3^ ± 1.502 × 10^−3^ M, and the chelating bond exhibited a greater affinity for hydrogen bonds and hydrophobic interactions [34,35,36]. Thermodynamic analysis further suggested that the interaction was driven primarily by hydrogen bonding and hydrophobic interactions, consistent with the observed exothermic behavior. Since it acts as a metal ion on the boundary between the hard and soft categories in Lewis acid classification, the ligands with chelating donor sites can be either nitrogen-containing or oxygen-containing compounds [37,38]. 

### 2.4. Assessment of Cell Proliferation and Viability in MC3T3-E1 Cells

**Figure 6:**
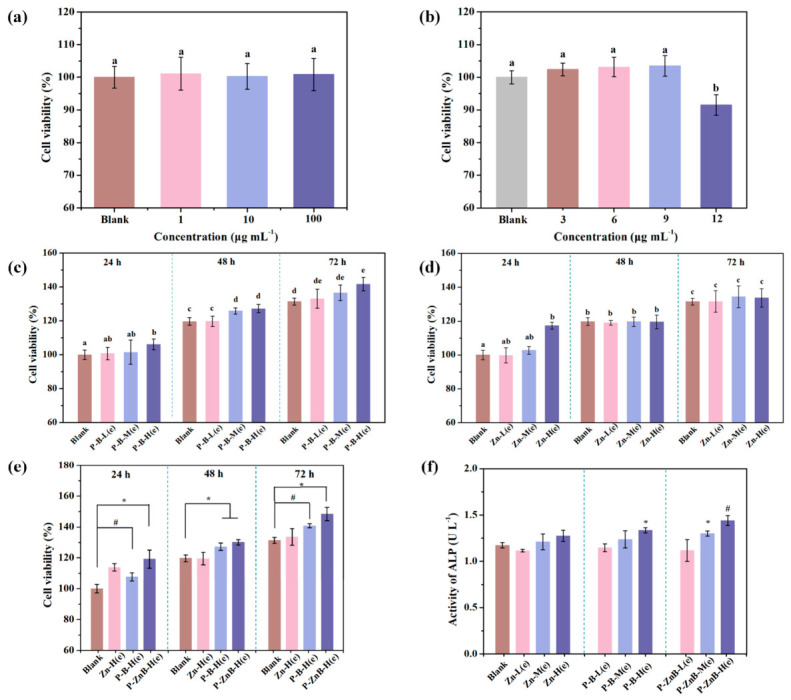
Proliferation and differentiation of MC3T3-E1 cells treated with different hydrogel extracts. (**a**) Cytotoxicity of H_3_BO_3_ after 24 h. (**b**) Cytotoxicity of Zn^2+^ after 24 h. (**c**–**e**) Cell proliferation after treatment with extracts for 24, 48, and 72 h. (**f**) Alkaline phosphatase (ALP) activity after 72 h. The extracts included Zn^2+^ alone (Zn-H(e), Zn-M(e), Zn-L(e)), P-2-CG with H_3_BO_3_ alone (P-B-H(e), P-B-M(e), P-B-L(e)), and P-2-CG with Zn^2+^ and H_3_BO_3_ (P-ZnB-H(e), P-ZnB-M(e), P-ZnB-L(e)). The final concentrations of each component are detailed in Table 1. Error bars represent standard deviation (*n* = 3). Groups with different letters (a, b, c, d, e) indicate significant differences at * *p* < 0.05. (#) 0.05 < *p* < 0.10 compared with the untreated blank group (one-way ANOVA with Tukey’s post hoc test). All extracts are designated with the suffix ‘(e)’.

Assessing the capacity for bone formation and cell survival is crucial when determining the suitability of peptide hydrogels as a bone adhesive substance [15,16]. It was confirmed that P-2-CG can promote the proliferation of osteoblasts in vitro [23]. To assess the cell proliferation and differentiation of hydrogel extracts (P-2-CG in different buffers), the MTT assay was performed on MC3T3-E1 cells. To prevent the damage from a higher concentration of buffer on cells, various concentrations of H_3_BO_3_ (0, 1, 10, and 100 μg mL^−1^) and ZnCl_2_ (0, 3, 6, 9, and 12 μg mL^−1^) were dissolved in the culture medium to access theviability rate of MC3T3-E1 cells in 2D cell cultures (Figure 6a,b) [17]. After the cells adhered to the bottom of the wells, they were cultured in different concentrations of hydrogel extracts (containing P-2-CG, ZnCl_2_, and H_3_BO_3_) for 24 h, 48 h, and 72 h, respectively. After culturing for 72 h, the groups treated with P-ZnB-H(e) (300 μg mL^−1^ P-2-CG in 9 μg mL^−1^ ZnCl_2_ and 18.93 μg mL^−1^ H_3_BO_3_) exhibited the highest proliferation activity at 144.72 ± 4.72% compared with untreated cells (131.38 ± 1.99%) (Figure 6e).

**Table 1:** Final concentrations of components in cell culture medium for each treatment group.

Group Code	P-2-CG (μg/mL)	ZnCl_2_ (μg/mL)	H_3_BO_3_ (μg/mL)
Zn-H(e)	–	9	–
Zn-M(e)	–	0.9	–
Zn-L(e)	–	0.09	–
P-B-H(e)	300	–	18.93
P-B-M(e)	30	–	1.893
P-B-L(e)	3	–	0.1893
P-ZnB-H(e)	300	9	18.93
P-ZnB-M(e)	30	0.9	1.893
P-ZnB-L(e)	3	0.09	0.1893

### 2.5. Expression Level Determination of Osteoblast Differentiation Markers

**Figure 7:**
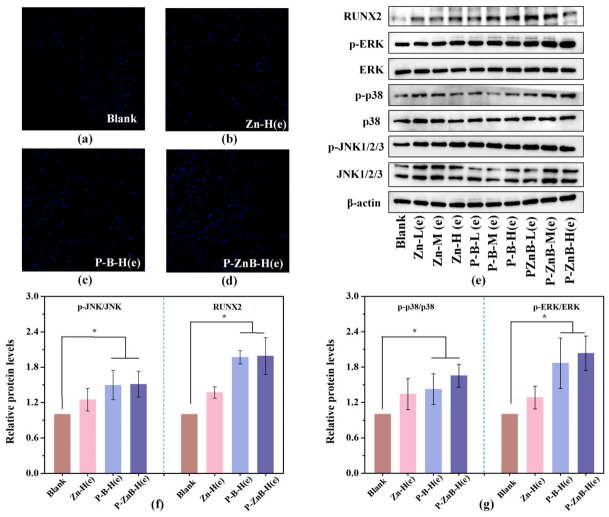
Effects of hydrogel extracts on the proliferation and differentiation of MC3T3-E1 cells. (**a**–**d**) Confocal laser scanning microscopy images of MC3T3-E1 cells stained with DAPI (blue, cell nuclei) after 72 h of treatment: (**a**) untreated control (blank); (**b**) Zn-H(e) (9 μg·mL^−1^ ZnCl_2_ alone); (**c**) P-B-H(e) (300 μg·mL^−1^ P-2-CG + 18.93 μg·mL^−1^ H_3_BO_3_); (**d**) P-ZnB-H(e) (300 μg·mL^−1^ P-2-CG + 9 μg·mL^−1^ ZnCl_2_ + 18.93 μg·mL^−1^ H_3_BO_3_). All extracts are designated with the suffix ‘(e)’. (**e**–**g**) Western blot analysis of MAPK pathway proteins. MC3T3-E1 cells were treated with the indicated extracts for 72 h, and the protein levels of p-p38/p38, p-ERK/ERK, and p-JNK1/2/3/JNK1/2/3 were quantified. Error bars represent standard deviation (*n* = 3). (*) *p* < 0.05 compared with the untreated blank group (one-way ANOVA with Tukey’s multiple comparison test).

The morphology and density of MC3T3-E1 cells after treatment were examined by confocal laser scanning microscopy following DAPI staining (blue, cell nuclei) [12,13]. As shown in Figure 7a–d, cells in the control group (untreated) exhibited normal nuclear morphology and moderate density (Figure 7a). Treatment with Zn-H(e) (9 μg·mL^−1^ ZnCl_2_ alone) resulted in slightly reduced cell density compared to control (Figure 7b). In contrast, cells cultured with P-B-H(e) (300 μg·mL^−1^ P-2-CG and 18.93 μg·mL^−1^ H_3_BO_3_) showed increased nuclear density and more uniform distribution (Figure 7c). The highest nuclear density and most pronounced cell spreading were observed in the P-ZnB-H(e) group (300 μg·mL^−1^ P-2-CG, 9 μg·mL^−1^ ZnCl_2_, and 18.93 μg·mL^−1^ H_3_BO_3_), indicating a marked increase in cell number and viability (Figure 7d). These observations suggest that the complete extract containing peptide, Zn^2+^, and boric acid promotes MC3T3-E1 cell proliferation in a concentration-dependent manner over 72 h. The control of cell multiplication in multicellular species is a multifaceted process, mainly governed by external growth factors supplied by neighboring cells. Within this context, the mitogen-activated protein kinase (MAPK) superfamilies are known to be pivotal in orchestrating various intricate cellular processes, including cell multiplication, differentiation, organismal development, malignant transformation, and programmed cell death. These signaling cascades operate by acting on molecular mechanisms that specifically target the MAPK pathway [39,40,41,42]. Moreover, recent studies on cell-encapsulating hydrogels have demonstrated that three-dimensional gel matrices can modulate intracellular signaling pathways in response to external stimuli, a concept that aligns with our observations [43]. To assess the possible osteogenic potential of hydrogel extracts, we further employed Western blot analysis to determine the expression levels of key phenotypic marker factors associated with preosteoblastic differentiation during bone formation process. Furthermore, the MAPK signaling cascade serves as a non-canonical alternative pathway, which activates the p38, ERK, and JNK1/2/3 signaling pathways and thereby enhances the expression of RUNX2. To investigate the regulatory effect of hydrogels on osteoblast differentiation, the expressions and phosphorylation statuses of p-38, ERK, and JNK1/2/3 were analyzed in the current research. To explore the underlying mechanism of hydrogel-mediated osteoblast regulation, the phosphorylation ratios of p-38, ERK, and JNK1/2/3 were analyzed in this research [41]. As shown in Figure 7e, P-ZnB-H(e) and P-B-H(e) increased the protein expression of p-p38/p38, p-ERK/ERK, and p-JNK1/2/3/JNK1/2/3. Additionally, alkaline phosphatase (ALP) activity, an important indicator of osteoblast differentiation, was measured to assess the functional status of osteoblasts in the culture system. The ALP activity was significantly increased when cultured in P-ZnB-H(e) for 72 h (1.43 ± 0.06 U L^−1^) in Figure 6f. P-Zn-9 hydrogels demonstrated favorable biocompatibility, facilitating the proliferation of MC3T3-E1 cells and holding potential applications in the field of bone repair.

### 2.6. Bone Regeneration Capacity

**Figure 8:**
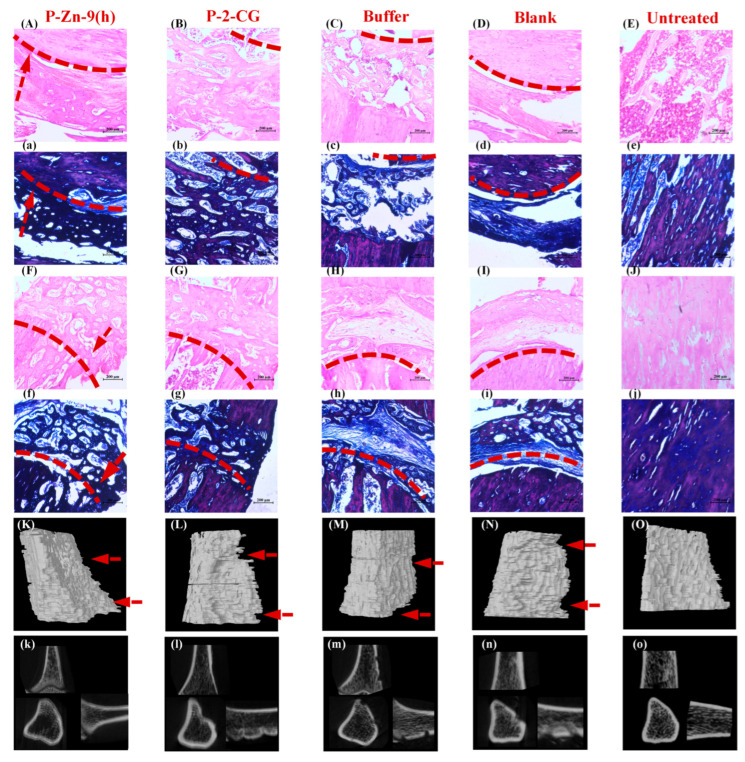
Representative histological H&E (**A**–**J**), Masson (**a**–**j**) staining, Micro-CT 3D reconstructed (**K**–**O**), and 2D rebuilding images (**k**–**o**) of the whole bone a between the hydrogel and the inner core of bones of sample-injected defect tibias after twenty-day treatment. New bone formation facilitated by P-Zn-9(h), P-Zn-0(h), buffer, blank, and the untreated samples. (**K**–**O**) Micro-CT 3D reconstructed models of new bone formation in SD rat tibias defects with different hydrogels injected and 2D rebuilding images of the newly formed bone within the defect area, after twenty-day treatment (*n* = 6).

To explore the capacity of hydrogels in facilitating in vivo bone repair, an animal model with critical-sized defects in mouse tibias was constructed. After a twenty-day observation period, the morphologies of the newly formed bones within the defects were assessed using X-ray micro-computed tomography (micro-CT). The reconstructed micro-CT 3D models showed that denser bone tissues were formed within the defects injected with P-Zn-9(h) (Figure 8K). Compared to the buffer and blank groups, the defects injected with P-Zn-9(h) (Figure 8A,F) and P-Zn-0(h) (Figure 8B,G) showed more extensive bone filling, whereas the buffer group (Figure 8C,H) exhibited moderate bone growth and the blank group (Figure 8D,I) showed minimal bone growth. The newly regenerated bone tissues were also evaluated histologically by Hematoxylin and Eosin (H&E) and Masson staining. As shown in Figure 8A–J, dotted lines in red indicate the inner core of the bone. Qualitative histological examination revealed that P-Zn-9(h) and P-Zn-0(h) exhibited greater bone tissue and osteoid staining compared to the buffer and blank groups. Additional new osteocytes were present in the newly formed bone tissues of P-Zn-9(h) and P-Zn-0(h), whereas new connective fibrous tissue formed compactly and densely between the hydrogel and the inner core of the bone in P-Zn-9(h) (Figure 8A,F). These findings suggest that P-Zn-9(h) may promote bone regeneration and ossification at the interface between the hydrogels and the defects. Furthermore, when compared to the control groups (buffer and blank injections), the P-Zn-9(h) and P-Zn-0(h) formulations appeared to show more compact and layered bone precursor tissues, whereas new bone formation in the buffer and blank injected groups was noticeably reduced. Given the qualitative nature of this assessment (without quantitative micro-CT indices such as BV/TV or BMD), these results should be considered preliminary. Nonetheless, they indicate that P-Zn-9(h) has promising biocompatibility and may support in vivo bone formation, suggesting its potential for future application in bone defect repair. To achieve this objective, the design of self-assembling supramolecular hydrogels derived from food-derived peptides was pursued, as these materials can offer bio-adhesive properties primarily arising from hydrogen bonding and hydrophilic interactions.

## 3. Conclusions

In this study, a functional dodecapeptide (P-2-CG) was designed to self-assemble into supramolecular hydrogels via Zn^2+^ chelation, without the need for additional crosslinking agents or chemical modifications. The Zn^2+^ concentration was found to critically modulate the microstructure of the hydrogels, with the P-Zn-9(h) formulation exhibiting a smooth, porous network with pore diameters of approximately 10 μm, which provided a favorable architecture for bio-adhesion and cell infiltration.

The following conclusions can be drawn:

(1) Qualitative evaluation of the P-Zn-9(h) revealed apparent bio-adhesive properties on bone tissue in an ex vivo porcine rib model, indicating its potential as an injectable bone adhesive material. Quantitative adhesion tests are required to substantiate this preliminary finding.
(2) In vitro assays showed that P-ZnB-H(e) significantly promoted the proliferation (144.72 ± 4.72% at 72 h) and differentiation (ALP activity 1.43 ± 0.06 U·L^−1^) of MC3T3-E1 osteoblasts. Western blot analysis revealed that these effects were associated with the activation of the MAPK signaling pathway (p-p38/p38, p-ERK/ERK, and p-JNK1/2/3/JNK1/2/3) and the upregulation of RUNX2 expression.
(3) In an in situ mouse tibia defect model, the P-Zn-9(h) facilitated outstanding osseointegration and bone regeneration, as confirmed by micro-CT and histological staining.

Despite these promising findings, several limitations should be acknowledged. The bio-adhesion test was qualitative, lacking standardized lap-shear strength measurements. Long-term biocompatibility and ISO-standard cytotoxicity validation are needed before clinical translation. Additionally, the exact chelation stoichiometry and the dynamic stability of the Zn^2+^-peptide coordination under physiological conditions warrant further investigation. Moreover, the in vivo bone defect study only evaluated a 20-day time point; long-term outcomes were not assessed, and no direct comparison with clinically established bone graft materials or commercial adhesives was performed. These aspects should be addressed in future studies.

Nevertheless, the present work establishes a straightforward and bio-inspired strategy for constructing self-assembling peptide hydrogels with intrinsic bio-adhesive and osteogenic activities. These materials offer imaginative opportunities for injectable bone repair systems and food-derived bio-adhesive platforms. Looking forward, the integration of machine learning into gel formulation and process optimization, as reviewed by Zhang et al., may facilitate the rational design of Zn^2+^-responsive self-assembling peptide hydrogels with tunable mechanical and bio-adhesive properties [44], paving the way for future developments in precision bone tissue engineering.

## 4. Materials and Methods

### 4.1. Materials and Chemicals

Deuterium oxide (D2O, 99.9%), potassium bromide (KBr), and 3-(4,5-dimethylthiazol-2-yl)-2,5-diphenyltetrazolium bromide (MTT) were sourced from Sigma-Aldrich Chemical Co., Ltd. (St Louis, MO, USA). Additionally, ZnCl_2_, boric acid (H_3_BO_3_), and dimethyl sulfoxide (DMSO) were sourced from Tianjin Damao Chemical Reagent Co., Ltd. (Tianjin, China). Ultrapure water was obtained using a Milli-Q Academic system (18.2 MΩ, MING-CHE 24V, Millipore, France). Additionally, a panel of antibodies was procured from ABclonal Biotech (Wuhan, China), including β-Actin (1/5000 dilution), runt-related transcription factor 2 (Runx 2) (1/1000 dilution), JUN N-terminal kinases 1/2/3 (JNK1/2/3) (1/1000 dilution), phosphorylated JNK1/2/3 (1/1000 dilution, targeting T183/T183/T221), p38 (1/1000 dilution), phosphorylated p38 (1/1000 dilution, targeting Y182), extracellular signal-regulated kinase (ERK) (1/1000 dilution), phosphorylated ERK (1/1000 dilution, targeting T183/T187), and HRP Goat anti-rabbit IgG (H+L) (1/5000 dilution). A specific peptide, designated P-2-CG, with the amino acid sequence IEELEEELEAER, was chemically synthesized by Shanghai Qiangyao Biotechnology Co., Ltd. in Shanghai, China. All other chemicals and reagents were of analytical or chromatographical grade.

### 4.2. Assembled Hydrogel Preparation

Zinc-chelated hydrogels formed by P-2-CG were prepared in three steps. The dodecapeptide P-2-CG (IEELEEELEAER) was dissolved in deionized water to 18 mM to form the peptide stock solution [17,24]. Buffer solutions containing 250 mM H_3_BO_3_ and ZnCl_2_ at 0, 6, 12, 18, or 24 mM were then prepared. Equal volumes (150 μL) of the peptide stock and each buffer solution were mixed and allowed to stand at 20 °C for at least 2 h, producing transparent, homogeneous hydrogels. The resulting final concentrations were 9 mM P-2-CG, 125 mM H_3_BO_3_, and ZnCl_2_ at 0, 3, 6, 9, or 12 mM. Hydrogels were labeled P-Zn-0(h), P-Zn-3(h), P-Zn-6(h), P-Zn-9(h), and P-Zn-12(h), where “h” denotes the hydrogel state.

### 4.3. 3D Dynamic Light Scattering Analysis

To conduct the analysis, a 3D Dynamic light scattering (3D DLS) measurement was carried out using a DLS system (LSI Company, Fribourg, Switzerland), which included an ALV light scattering device. This equipment was equipped with a 400 mW argon ion laser that operates at a wavelength of 532 nm. In the context of particle size analysis, Cui et al. applied the Stokes-Einstein equation along with intensity cross-correlation function to determine the particle hydrodynamic radius, which proved effective for both quasi-spherical and irregularly shaped particles [30]. Conversely, traditional light scattering methods require the measurement and analysis of only single scattered light, making them unsuitable for concentrated solutions where multiple scattering occurs [30]. To address this limitation, P-2-CG and zinc chelated hydrogel samples including P-Zn-3(h), P-Zn-6(h), P-Zn-9(h), P-Zn-12(h) were prepared by diluting them fifteen times with deionized water, followed by filling 200 µL of the diluted solutions into 5 mm-path-length cuvettes. In dynamic light scattering analysis, the intercept intensity g^2^(t) of the correlation function significantly impacts the measurement’s accuracy and precision because it plays a crucial part in fitting models to the collected data. All measurements were conducted at 20 °C Celsius, and g^2^(t) values were recorded at various time intervals within a 90 °C detection angle. Each measurement process lasted a total of 3600 s, during which the intensity cross-correlation functions were obtained from the intensities of the scattered light directly recorded. The intensity g^2^(t) was obtained from the intensities of the scattered light directly recorded. The CONTIN algorithm was applied to analyze the intensity cross-correlation functions. The intensity cross-correlation function and field cross-correlation function were calculated as follows:

(1) $$Intensity\;\text{cross-correlation}\;function:g^{\left(2\right)}(t)=\frac{{<\frac{I\left(0\right)}{I(t)}>}^{t}}{{I(t)}^{2}}$$

(2) $$G^{\left(2\right)}(t)=\underset{t\to \infty }{\lim}\frac{1}{2t}\int_{-t}^{t}I\left(t^{\prime }\right)*I(t^{\prime }+t)dt^{\prime }$$

(3) $$Field\;\text{cross-correlation}\;function:g^{\left(1\right)}(t)={<g^{\left(2\right)}\left(t\right)-1>}^{0.5}$$

### 4.4. Bio-Adhesion of Porcine Ribs

Once the hydrogel had solidified, a qualitative assessment was conducted to evaluate the capability of the zinc-chelated hydrogel in adhering to actual osseous structures, aiming to mimic the process of bone attachment [45]. Twenty-six porcine rib bones were collected, then cut with a surgical blade, and their intersecting surfaces as well as weights were smoothed to facilitate subsequent comparisons. Subsequently, the rib bones were positioned vertically, with samples P-Zn-0(h), P-Zn-3(h), and P-Zn-9(h) applied to their intersecting surfaces to ensure the two halves were uniformly and completely bonded across the overlapping region. After a 5 min period of applying stress, the bones were carefully lifted out. Images were captured to record the adhesive performance as a qualitative indicator. Quantitative adhesion strength measurements are required in future studies to validate these observations.

### 4.5. Morphology Analysis

A small volume of the test solutions including P-Zn-3(h), P-Zn-6(h), P-Zn-9(h), and P-Zn-12(h) were dropped onto carbon-coated copper grids (400 mesh, Plano brand, Plano, IL, USA) for the preparation of TEM samples [46]. After a 5 min period of adsorption, the sample was stained using 2% uranyl acetate for a duration of 2 min, following which the excessive solution was carefully removed with filter paper. Subsequently, the treated specimens were loaded into a cryo-transfer holder (CT3500, Gatan, Munich, Germany) and subsequently introduced into a cryogenic-transmission electron microscope (Cryo-TEM) system (Hitachi H-7650, Hitachi, Tokyo, Japan) operating at 200 kV [47]. 

To conduct cryogenic-scanning electron microscopy (Cryo-SEM) analysis (using a Hitachi S8020 field emission SEM from Hitachi High-technologies Co., Tokyo, Japan) [48], samples including P-Zn-0(h), P-Zn-3(h), P-Zn-6(h), P-Zn-9(h), and P-Zn-12(h) were prepared by first freezing them promptly in liquid nitrogen before transferring them into the cryo-preparation chamber where they were sectioned with a freezing knife. Subsequently, these specimens were rapidly frozen in liquid nitrogen before being transferred into a cryo-preparation chamber, where a freezing knife was used to section them. Subsequently, the freshly exposed sections of the specimens underwent sublimation at −90 °C, during which moisture within the samples was extracted to a depth of several micrometers, effectively covering the structural details beneath the newly formed surface layer. Following the sublimation process, the samples underwent sputter-coating with gold before being transferred into the SEM chamber. The analysis was performed at a temperature of −145 °C and an acceleration voltage set at 5.0 kV.

### 4.6. NMR and FT-IR Spectroscopic Analysis

NMR experiments were conducted on a 700 MHz Bruker AVANCE III spectrometer (Bruker, Billerica, MA, USA) located at the Beijing NMR Center, operating at 298 Kelvin. The resulting NMR datasets were processed using TOPSPIN 3.6.3 software (Bruker, Billerica, MA, USA) and subsequently analyzed through the Sparky program [49,50]. Recent studies have also utilized NMR relaxometry to characterize the molecular mobility and structural integrity of alginate-based gel systems [51]. For the hydrogels formed by P-2-CG, deuterium lock was achieved using 10% D2O (99.9%) and DSS was employed as the signal reference. To further explore the structural characteristics of the hydrogels, one-dimensional NMR spectroscopy was employed. To further characterize the hydrogels, Fourier transform infrared spectrometry (FTIR) analysis was conducted on a Thermo Scientific Nicolet iS5 (Thermo Scientific, Waltham, MA, USA) under room temperature conditions. The analysis utilized a wave number range spanning from 4000 cm^−1^ to 400 cm^−1^, with a total of 32 scans performed. To prepare the samples for analysis, hydrogels were first subjected to complete lyophilization using a vacuum freeze dryer. Following this process, the dried materials were ground into a fine powder and then mixed with KBr at a ratio of 1:100 before being compressed into tablets.

### 4.7. Isothermal Titration Calorimetry Analysis

Isothermal titration calorimetry (ITC) represents an extremely sensitive and robust technique for investigating the thermodynamics of molecular interactions [52]. All such ITC analyses were conducted at 25 °C by utilizing an Affinity ITC isothermal titration calorimeter (TA, Lindon, UT, USA). The investigation involved a non-simple interaction between metallic ions and the peptide, with the metal ion solution prepared to a final concentration in a buffer that included 20 mM ZnCl_2_ at pH 5.0. The peptide solution, which was prepared with a concentration of 0.5 mM and adjusted to pH 5.0, was the medium in which the metal ion binding reaction occurred. In the experiment, 350 μL of the P-2-CG solution (with a concentration of 0.5 mM and pH 5.0) was titrated by adding ZnCl_2_ through a series of 40 injections. Each injection had a volume of 80 μL, and the volume of each individual injection was 2.0 μL. During the titration process, the stirring speed was maintained at 750 rpm. Between successive injections, there was a 240 s interval. The data were then analyzed using the TA NanoAnalyze software (version 3.9.0, TA Instruments, New Castle, DE, USA), applying the “Independent Model” which assumed the presence of binding sites with identical binding characteristics, while not considering speciation. All isothermal titration calorimetry (ITC) measurements were initiated once a consistent baseline of 100 s had been established. To guarantee the reliability of the results, every peptide underwent titration on three separate occasions.

### 4.8. Cell Viability Assay

To assess cell viability, an MTT-based method was employed, using MC3T3-E1 cells (obtained from the Shanghai Cell Bank of the Chinese Academy of Sciences), which are derived from the calvaria of newborn mice. These cells were evaluated by exposing them to hydrogel extracts for the purpose of the test. The culture medium employed for these cells is α-Minimum Essential Medium (Hyclone, Logan, UT, USA), supplemented with 1% penicillin-streptomycin (Hyclone, Logan, UT, USA) and 10% fetal bovine serum (PAN-Biotech, Wimborne, UK). Generally, MC3T3-E1 cells were dispersed into cell growth medium and then plated into 96-well microtiter plates at a seeding density of 0.5 × 10^4^ cells per well, followed by a 24 h cultivation period at 37 °C in a 5% CO_2_ atmosphere to form a confluent cell monolayer. The hydrogel extracts were diluted in culture medium to obtain the following groups: Zn^2+^ alone (3 concentrations), P-2-CG + H_3_BO_3_ alone (3 concentrations), and P-2-CG + ZnCl_2_ + H_3_BO_3_ (3 concentrations). The final concentrations of each component in the culture medium are summarized in Table 1, followed by further incubation for 24, 48, and 72 h. Following this incubation period, the hydrogel-containing discs were rinsed three times with phosphate-buffered saline (PBS) for 5 min each, after which they were stained with 4′,6-diamidino-2-phenylindole (DAPI) for a duration of 5 min. Following the staining process, the discs were then observed and photographed using a confocal laser scanning microscopy (CLSM) on a Leica DMi-8 upright microscope with Leica TCS SP-8 confocal laser scanning head mounted (Leica Microsystems Inc., Wetzlar, Germany) to examine the cells within the hydrogel extracts that had been incubated for 72 h. After the incubation period, the medium containing the hydrogel extracts was removed, and the adherent cells were then exposed to a MTT solution (0.5 mg/mL concentration, 10 μL volume) for an additional 4 h cultivation. Subsequently, the culture medium was replaced by DMSO (150) to dissolve the formazan crystals, and the absorbance of the resulting DMSO solution at 490 nm was measured using a microplate reader (InfiniteTM M200, TECANE, Männedorf, Switzerland) [22,23]. All cell viability data were normalized to the 24 h untreated control (Blank) group, which was set as 100% at each time point. The Blank group exhibited gradual proliferation over 72 h, resulting in >100% viability at later time points; therefore, treatment groups were compared to the corresponding time-matched baseline rather than a fixed initial value. Alkaline phosphatase (ALP), an enzyme often used as a marker for the early stages of osteoblast differentiation, exhibits elevated levels in cell cultures during their growth phase. Following a 72 h cultivation period, the activity of this enzyme in the cell samples was quantitatively measured in accordance with the operational guidelines provided by the alkaline phosphatase activity assay kit (Beyotime, Haimen, China).

### 4.9. Western Blotting

MC3T3-E1 cells were suspended in cell culture medium and seeded into 6-well plates at a density of 1.0 × 10^5^ cells per well, being incubated for 72 h at 37 °C in 5% CO_2_. Subsequently, the collected cells were lysed using RIPA buffer containing PMSF to extract total cell proteins. Subsequently, the amount of protein in the extracted samples was quantified via the BCA protein assay method, and an aliquot of 20 μg total cell protein was selected for subsequent analysis. Subsequently, the separated proteins from the polyacrylamide gels were transferred onto polyvinylidene difluoride (PVDF) membranes through a transfer process. After the transfer, the PVDF membranes were blocked for 1 h at room temperature. Subsequently, the membranes were incubated with primary antibodies against β-actin (1:5000), Runx2 (1:1000), JNK1/2/3 (1:1000), phosphorylated JNK1/2/3 (1:1000, targeting T183/T183/T221), p38 (1:1000), phosphorylated p38 (1:1000, targeting Y182), ERK (1:1000), and phosphorylated ERK (1:1000, targeting T183/T187) overnight at 4 °C. After washing three times with TBST buffer, the membranes were then incubated with HRP-conjugated goat anti-rabbit IgG (H+L) secondary antibody at a dilution of 1:5000 for 2 h at room temperature. Subsequently, the membranes were subjected to three washes using TBST buffer. Subsequently, the gel was subjected to chromogenic reaction using a chemiluminescence detection kit (BeyoECL Plus, Beyotime, Shanghai, China) within a gel image processing system (MF-ChemiBIS 2.0, DNR, Jerusalem, Israel) [20,22]. After obtaining the gel images via the system, the molecular weight and net optical density of the target bands were analyzed using Image J 1.51 software.

### 4.10. Bone Injury Surgery

To assess the osteogenic potential of various tibias defect models, experiments were conducted in compliance with the guidelines set by the Animal Ethics Committee of Dalian Polytechnic University. Ten-week-old female Sprague-Dawley mice, weighing between 220 g and 250 g, supplied by Liaoning Changsheng Biotechnology Co., Ltd. (Benxi, China), were employed for this evaluation. (Certificate No: SCKK Liao 2015-0001). The relevant animal experiment design was carried out in accordance with the guidance of the National Institutes of Health Animal Laboratory. In the study, all Sprague-Dawley mice employed were subjected to standardized procedures that strictly adhered to the ethical guidelines established by the Ethics Committee of Dalian University of Technology (approval number: SYXK2017-0005). Subsequently, all mice were randomly assigned into five distinct groups to construct the P-Zn-9(h), P-Zn-0(h), buffer, blank (H_2_O), and untreated models. After inducing anesthesia via intraperitoneal administration of ethyl carbamate (20%, *w*/*v*), 2 mm deep holes were created using a drill with an outer diameter of 4.0 mm and an inner diameter of 3.0 mm in the distal portion of the tibias (right legs) [4,23]. During this process, physiological saline was sprayed on the surgical site to lower the temperature and minimize tissue damage. For the in situ defect analysis, P-Zn-9(h), P-Zn-0(h), buffer solution, and blank control (distilled water) were introduced, the inner core bones were then placed into the prepared holes, and subsequently, 5 microliters of various test samples were injected into each of these holes. Following a brief staining period, the tissues were carefully sutured layer by layer without delay.

### 4.11. Osteogenesis Evaluation

Following respective surgical procedures, specimens were harvested at 10 and 20 days post-operation, and then subjected to micro-computed tomography (SKYSCAN1272, Bruker, Karlsruhe, Germany) scanning for the purpose of assessing osseointegration between the implanted core bone and the surrounding host bone tissue. Before conducting the micro-CT assessment, the mice were put under anesthesia by combining oxygen with isoflurane, with an oxygen flow rate maintained at 0.2 mL min^−1^ [4]. During the imaging process, the following operational settings were applied: X-ray voltage, 50 kV; Filter, 0.5 mm aluminum. In the process, the region of interest (ROI) was identified on the cross-sectional images generated from the instrument’s scan data, and subsequent three-dimensional reconstruction was carried out by applying a thresholding method via the Evaluation V6.5-3 software [53]. Following the above steps, 34 mouse tibias were chosen, with muscle tissues removed to the greatest extent possible. Subsequently, the extracted tibias underwent a decalcification process by immersion in hydrochloric acid solution for a duration of 2 days, which effectively dissolved the calcium within the bone matrix, thereby softening the tissue to facilitate subsequent slicing procedures. Before preparing the samples, bones were preserved in 4% paraformaldehyde and then embedded using a Leica EG1150H paraffin embedding system (Leica Biosystems, Nussloch, Germany). Subsequently, each tibia’s longitudinal section was sliced into thin layers using a Leica RM2245 rotary microtome (Leica Microsystems, Nussloch, Germany). Subsequently, the thickness of these slices was set to 5–6 μm through adjustment. Subsequently, the histopathological assessment of the newly formed bone tissues was conducted using both Hematoxylin–Eosin (H&E) and Masson staining methods. After dewaxing treatment, mouse specimens were stained with Hematoxylin–Eosin method. Subsequently, the stained specimens were examined under an optical microscope (TI-DH, Nikon, Tokyo, Japan) [54]. 

### 4.12. Statistical Analysis

The samples were analyzed using isothermal titration calorimetry (ITC), with each sample undergoing three separate measurements to ensure data reliability, resulting in triplicate determinations for each experimental point. The one-way ANOVA and the HSD Tukey test were applied to evaluate the mean ± standard deviations (SD) of the three data sets, which were processed using SPSS software (SPSS 16.0 Inc., Chicago, IL, USA). A value of *p* < 0.05 was considered significant. Data visualization and figure preparation were carried out with the assistance of Origin 8.5 (Origin Lab Corporation, Northampton, MA, USA).

## Data Availability

The original contributions presented in this study are included in the article. Further inquiries can be directed to the corresponding author.
